# Predisposing factors for Hoffa’s fat pad syndrome: a systematic review

**DOI:** 10.1186/s43019-023-00192-4

**Published:** 2023-06-09

**Authors:** Diego Agustín Abelleyra Lastoria, Clerin Kulangara Benny, Caroline Blanca Hing

**Affiliations:** 1 St George’s University London, St George’s University Hospitals NHS Foundation Trust, London, SW17 0RE UK; 2 Medical University of Sofia, Sofia, Bulgaria; 3 Department of Trauma and Orthopaedics, St George’s University Hospitals NHS Foundation Trust, London, UK

**Keywords:** Hoffa’s fat pad syndrome, Risk factors, Systematic review

## Abstract

**Background:**

Hoffa’s fat pad syndrome has been defined as impingement of Hoffa’s fat pad, leading to oedema and fibrosis. The primary aim of this systematic review was to identify morphological differences in Hoffa’s fat pad between patients with and without Hoffa’s fat pad syndrome, evaluating them as risk factors predisposing to its development. The secondary aim was to summarize and evaluate current evidence pertaining to the management of Hoffa’s fat pad syndrome.

**Materials and Methods:**

The protocol for this review was prospectively registered (PROSPERO registration: CRD42022357036). Electronic databases, currently registered studies, conference proceedings and the reference lists of included studies were searched. All studies evaluating differences in Hoffa’s fat pad anatomy under imaging between patients with and without Hoffa’s fat pad syndrome were included, as well as those exploring epidemiological factors predisposing to its development (ethnicity, employment status, sex, age and BMI), and studies reporting on the effect of treatment on Hoffa’s fat pad morphology.

**Results:**

A total of 3871 records were screened. Twenty one articles satisfied the inclusion criteria, evaluating 3603 knees of 3518 patients. Patella alta, increased tibial tubercle-tibial groove distance, and increased trochlear angle were found to predispose the development of Hoffa’s fat pad syndrome. Trochlear inclination, sulcus angle, patient age and BMI were not associated with this condition. The link between Hoffa’s fat pad syndrome and ethnicity, employment, patellar alignment, Hoffa’s fat pad composition, physical activity and other pathological processes cannot be established due to lack of evidence. No studies reporting on treatment for Hoffa’s fat pad syndrome were identified. Though weight loss and gene therapy may provide symptomatic relief, further research is required to corroborate these claims.

**Conclusion:**

Current evidence suggests that high patellar height, TT-TG distance, and trochlear angle predispose the development of Hoffa’s fat pad syndrome. In addition, trochlear inclination, sulcus angle, patient age and BMI do not seem to be associated with this condition. Further research should explore the link between Hoffa’s fat pad syndrome and sport as well as other conditions pertaining to the knee. In addition, further study evaluating treatment approaches for Hoffa’s fat pad syndrome is required.

## Background

Hoffa’s fat pad is located inferior to the patella. It lies posterior to the patellar tendon and anterior to the femoral condyles. It is an intra-capsular, extra-synovial structure [1]. Some of its proposed functions include fluid secretion to promote efficient lubrication of the knee joint, occupation of joint dead space to maintain its integrity, and reducing friction between the femoral condyles and joint capsule [1].

Hoffa’s fat pad syndrome has been defined as the impingement of Hoffa’s fat pad, occurring with concomitant pathology, leading to its oedema and fibrosis [2]. It can also be caused by repetitive micro trauma resulting in inflammation and fibrous changes to Hoffa’s fat pad [3]. Inflammation of Hoffa’s fat pad could lead to the synovial membrane being compressed against the femoral condyles, resulting in effusions, anterior knee pain, and functional impairment [1].

Treatment for Hoffa’s fat pad syndrome involves commencing with conservative approaches, such as physical therapy, taping, and muscle training. These can be complemented with injections of local anaesthetic and/or corticosteroids [4]. Surgical treatments are recommended if non-operative approaches fail to provide symptomatic relief. These include fat pad excision, synovectomy, and denervation of the inferior pole of the patella [4].

Possible risk factors for developing Hoffa’s fat pad syndrome have been proposed. Examples include lateral patellar displacement [5] and practising sports at a high level [6]. A meta-analysis evaluated parameters of patellofemoral mal-tracking associated with superolateral Hoffa’s fat pad oedema. Large patellar tilt, lateralization, tibial tuberosity-trochlear groove (TT-TG) distance, and high patellar height were associated with superolateral Hoffa’s fat pad oedema [7]. Factors other than those pertaining to patellar mal-tracking must be explored. Two recent systematic reviews on Hoffa’s fat pad syndrome reported on its management [8, 9]. These did not comment on factors predisposing the development of this condition. Knowledge of risk factors for Hoffa’s fat pad syndrome could help explain its pathophysiological processes, allowing for specific and effective treatment approaches to be designed, and must therefore be explored. In addition, new potential treatments have been described since the performance of the aforementioned reviews, such as stem cell therapies [10, 11]. The identification of studies published since, may provide further insight into the treatment of Hoffa’s fat pad syndrome. The effectiveness and feasibility of new therapies must be evaluated.

The primary aim of this systematic review was to identify morphological differences in Hoffa’s fat pad between patients with and without Hoffa’s fat pad syndrome, evaluating them as risk factors predisposing to its development. The secondary aim was to summarize and evaluate current evidence pertaining to the management of Hoffa’s fat pad syndrome.

## Methods

The PRISMA 2020 checklist was followed [12]. The protocol for this review was prospectively registered on PROSPERO with registration number CRD42022357036.

### Study eligibility

All studies evaluating morphological and epidemiological factors predisposing to the development of Hoffa’s fat pad syndrome were included. Morphological factors entailed differences in Hoffa’s fat pad anatomy under imaging between patients with and without Hoffa’s fat pad syndrome. Epidemiological factors evaluated were ethnicity, employment status, sex, age and BMI. In addition, studies reporting on the effect of treatment on Hoffa’s fat pad morphology were included.

Papers not reporting original data such as literature or systematic reviews were excluded, along with case reports, animal studies and letters to the editor. Theoretical models, studies not evaluating potential risk factors for Hoffa’s fat pad syndrome, and those not reporting on the effect of treatment on Hoffa’s fat pad morphology were also excluded. There were no constraints based on language or publication status.

### Search strategy and data extraction

Database search and data extraction were conducted independently by the first and second authors. Searches were conducted twice for quality assurance. The first search was conducted on 13/09/2022. The search was repeated on 29/01/2023. The search strategy is attached (Appendix 1). We searched the following electronic databases via OVID from 01/01/2012 to capture articles published in the last 10 years, identifying the latest published literature: MEDLINE, Global Health, and Embase. Currently registered studies were reviewed using the databases ISRCTN registry, the National Institute for Health Research Portfolio, the UK National Research Register Archive, the WHO International Clinical Trials Registry Platform, and OpenSIGLE (system for information on grey literature in Europe). Conference proceedings from the European federation of national associations of orthopaedics and traumatology (EFORT), British Orthopaedic Association and British Trauma Society were searched. The reference lists of included studies were also searched.

### Methodological appraisal

Level of evidence and risk of bias of each study included were evaluated independently by the first and second authors. The level of evidence of the studies presented was determined with the March 2009 Oxford Centre for Evidence-Based Medicine: Levels of Evidence [13]. The anatomical quality assessment (AQUA) tool was used to assess the risk of bias of non-interventional anatomical studies [14]. The Cochrane Collaboration’s risk of bias tool was used to assess risk of bias in randomized controlled trials (RCTs) [15].

## Results

**Fig. 1:**
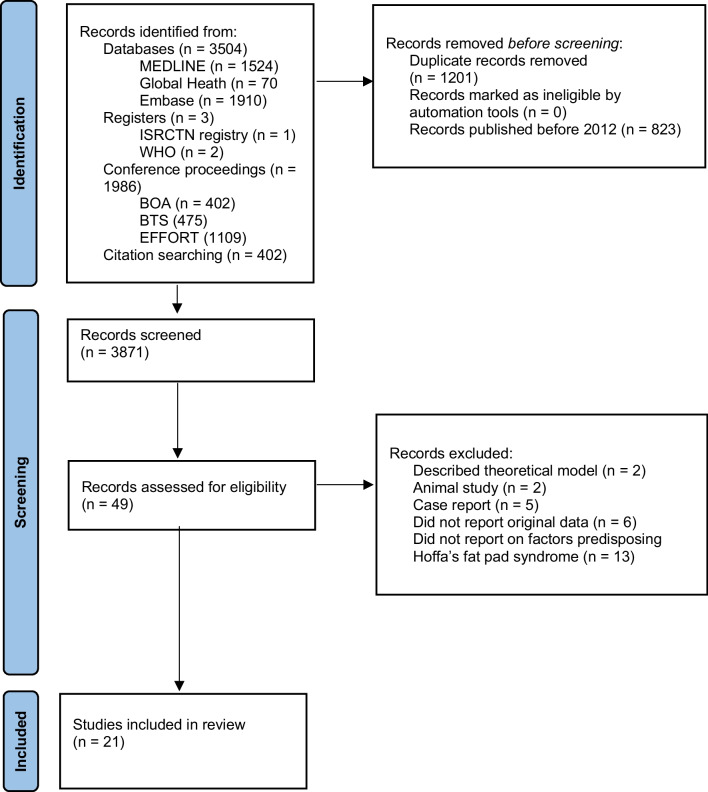
PRISMA diagram depicting the study collection process

A total of 3871 records were screened, with 49 potentially eligible articles identified (Fig. 1). Twenty-eight were excluded on the basis of the pre-specified exclusion criteria. A total of 21 studies were included, evaluating 3603 knees of 3518 patients (Table 1). Quantitative pooled analysis was prevented by the heterogeneity of the data in terms of criteria for Hoffa’s fat pad syndrome and reporting of outcomes. Therefore, a narrative synthesis was performed.

**Table 1:** Risk of bias and population characteristics of studies included in this review

Study	Study design, level of evidence	Risk of bias	Imaging modality	Parameters evaluated	Patient groups	Number of patients (males, females)	Number of knees	Mean patient age (years)
Zhong et al., 2022	Non-interventional anatomical study, 4	Low	Hydrogen proton MR spectroscopy	Hoffa’s fat pad fat fraction, unsaturation index	Patients with osteoarthritis	48 (16, 32)	64	55
Yu, 2022	Non-interventional anatomical study, 4	High	MRI	Hoffa’s fat pad oedema following football-related injury	Group 1: football-related knee injuries Group 2: outpatients with acute knee injury	Group 1: 29 (29, 0) Group 2: 31 (31, 0)	Group 1: 31 Group 2: 31	Group 1: 23.6 Group 2: Range 18–30
von Engelhardt et al., 2020	Non-interventional anatomical study, 4	Low	MRI	Hoffa’s fat pad dimensions, oedema and fibrosis	Group 1: Hoffa’s fat pad impingement Group 2: patients with other knee pathologies, not including Hoffa’s fat pad impingement	Group 1: 62 (32. 30) Group 2: 255 (164, 91)	Group 1: 62 Group 2: 255	Group 1: 48 Group 2: 40
Kim et al., 2022	Non-interventional anatomical study, 4	High	MRI	PPTA, patient sex, age, BMI	Group 1: Hoffa’s fat pad syndrome Group 2: medial patellar plica syndrome Group 3: chondromalacia patella	Group 1: 26 (23,3) Group 2: 86 (72, 14) Group 3: 44 (39, 5)	Group 1: 26 Group 2: 86 Group 3: 44	Group 1: 30.8 Group 2: 29.2 Group 3: 31.5
Cilengir et al., 2021	Non-interventional anatomical study, 4	NFT	MRI	Lateral patellar tilt angle	Group 1: lateral patellar tilt angle > 5° Group 2: lateral patellar tilt angle < 5°	Group 1: 406 Group 2: 40	446	NR
Delorme and Jibri, 2021	Non-interventional anatomical study, 4	NFT	MRI	Relationship between Hoffa’s fat pad impingement and patellar tendinosis	Group 1: patellar tendinosis Group 2: control group	Group 1: 94 Group 2: 94	188	NR
Xiaolong et al., 2021	Non-interventional anatomical study, 4	NFT	MRI	ISR, trochlear angle	Superolateral Hoffa’s fat pad oedema	60	60	NR
Kim et al., 2020	Non-interventional anatomical study, 4	Low	MRI	Cross sectional area, PPTA, ISR, sulcus angle, trochlear inclination, TT-TG distance, patellar alignment and tilt	Group 1: Hoffa’s fat pad syndrome Group 2: control group without knee pathology	Group 1: 46 (29, 17) Group 2: 39 (29, 10)	Group 1: 44 Group 2: 78	Group 1: 29.2 Group 2: 31.3
Kim et al., 2019	Non-interventional anatomical study, 4	NFT	MRI	Sulcus angle, ISR, TT-TG distance, patellar alignment and tilt	Group 1: superolateral Hoffa’s fat pad oedema Group 2: patients without superolateral Hoffa’s fat pad oedema	68	Group 1: 24 Group 2: 47	NR
Campagna et al., 2012	Non-interventional anatomical study, 4	NFT	MRI	ISR, age, distance between patellar ligament and lateral trochlear facet	Group 1: superolateral Hoffa’s fat pad oedema Group 2: patients without superolateral Hoffa’s fat pad oedema	Group 1: 30 Group 2: 60	90	NR
Widjajahakim et al., 2017	Non-interventional anatomical study, 4	Low	MRI	ISR, trochlear angle, sulcus angle, trochlear inclination, TT-TG distance, bisect offset	Patients with (152) or without (982) Hoffa’s fat pad oedema	1134 (421, 713)	1134	66.8
Mikkilineni et al., 2018	Non-interventional anatomical study, 4	NFT	Ultrasound	Diameter of largest vessel supplying Hoffa’s fat pad, compressibility, vascularity, motion	Group 1: Hoffa’s fat pad impingement Group 2: asymptomatic controls	Group 1: 11 Group 2: 10	Group 1: 11 Group 2: 10	NR
Gürsoy et al., 2018	Non-interventional anatomical study, 4	Some concerns	MRI	ISR, trochlear angle, patellofemoral angle	Group 1: superolateral Hoffa’s fat pad oedema Group 2: patients without superolateral Hoffa’s fat pad oedema	Group 1: 50 (17, 33) Group 2: 50 (28, 22)	Group 1: 50 Group 2: 50	Group 1: 38.6 Group 2: 26.3
Mehta et al., 2015	Non-interventional anatomical study, 4	Low	MRI	Sulcus angle, TT-TG distance, lateral trochlear inclination, patellar translation, lateral patellar displacement, lateral patellar tilt, trochlear depth	Group 1: superolateral Hoffa’s fat pad oedema Group 2: normal knees	Group 1: 8 (0, 8) Group 2: 8 (0, 8)	Group 1: 16 Group 2: 16	19.9
Matcuk et al., 2014	Non-interventional anatomical study, 4	High	MRI	TT-TG distance, ISR, sulcus angle, lateral trochlear inclination, length of medial patellar facet, patellar angle, patellofemoral angle, patellar length, trochlear cartilage overlap, trochlear cartilage index. length of medial and lateral trochlea, trochlear depth, lateral patellar displacement and tilt, ventral trochlear prominence, length of lateral facet of the patella, largest medial and lateral AP diameters of the femur, sulcus height and patellar cartilage length	Group 1: superolateral Hoffa’s fat pad oedema Group 2: normal knees	Group 1: 65 (24, 41) Group 2: 40 (17, 23)	Group 1: 71 Group 2: 45	Group 1: 34 Group 2: 28
Jibri et al., 2012	Non-interventional anatomical study, 4	Low	MRI	ISR, TT-TG, trochlear depth, patellar translation, patellofemoral angle, lateral patellar displacement and lateral patellar tilt	Group 1: superolateral Hoffa’s fat pad oedema Group 2: patients without superolateral Hoffa’s fat pad oedema	Group 1: 100 (24, 76) Group 2: 100 (37, 63)	Group 1: 100 Group 2: 100	Group 1: 31 Group 2: 33
van Middelkoop et al., 2018	Non-interventional anatomical study, 4	Low	MRI	ISR, sulcus angle, patellar tilt, translation, patellar cartilage overlap, Wiberg classification, and bisect offset	Patients with patellofemoral pain	133 (55, 78)	133	30.2
Kitagawa et al., 2022	2b, low-quality RCT	High	Ultrasound	Effect of manual therapy or hot pack treatment on the flexibility of Hoffa’s fat pad	Group 1: manual therapy Group 2: hot pack application Group 3: control group (relaxed limbs)	Group 1: 21 Group 2: 22 Group 3: 21	Group 1: 21 Group 2: 22 Group 3: 21	20.7
Pogacnik Murillo et al., 2017	1b, RCT	Some concerns	MRI	Effect of diet and/or exercise on Hoffa’s fat pad volume, surface area, and thickness	Group 1: exercise Group 2: diet-induced weight loss Group 3: diet-induced weight loss + exercise	Group 1: 36 (9, 27) Group 2: 35 (11, 24) Group 3: 35 (9, 26)	Group 1: 36 Group 2: 35 Group 3: 35	NR
Steidle-Kloc et al., 2015	4, prospective interventional study	NFT	MRI	Effect of weight gain or weight loss on Hoffa’s fat pad volume	Group 1: patients with 20% weight gain Group 2: patients with 20% weight loss	Group 1: 10 (4, 6) Group 2: 9 (1, 8)	Group 1: 10 Group 2: 9	NR
Kalsi et al., 2018	RCT	NFT	MRI	Effect of Tissue-Gene-C on Hoffa’s fat pad synovitis and effusion-synovitis	Group 1: Tissue Gene-C (a TGF-Beta 1 expression vector) Group 2: placebo control	Group 1: 68 Group 2: 34	Group 1: 68 Group 2: 34	NR

*MRI* magnetic resonance imaging, *MR* magnetic resonance, *NFT* non-full text study, *NR* not reported, *PPTA* patella-patellar tendon angle, *BMI* body mass index, *ISR* Insall-Salvati ratio, *TT-TG* tibial tubercle-tibial groove, *AP* anteroposterior

### Study quality assessment

The findings of the study quality assessment are presented in Table 1. There were 17 non-interventional anatomical studies (evidence level 4), one prospective interventional study (evidence level 4), and three RCTs. One of the RCTs was of low-quality, and was assigned an evidence level 2b [16]. The other RCT had an evidence level 1b. Level of evidence of the remaining RCT could not be assessed due to this being a non-full text study [17]. Of the 21 studies included, eight were non-full text studies, preventing the performance of a risk of bias assessment. The study by Gürsoy et al. [18] exhibited some concerns regarding its risk of bias, including patient groups differing in age, with this confounder unaccounted for. There were some concerns regarding risk of bias in the study by Pogacnik Murillo et al. [19], since it was unclear if data was analysed in accordance with a pre-specified analysis plan.

Four studies were deemed to carry a high risk of bias. Kim et al. [20] grouped three groups of patients with different knee pathologies and compared them to a control group of normal knees, rather than making a separate direct comparison between each pathology and the control group. Yu et al. [21] did not present complete results and information on patient demographics, and presented results in an unclear manner. Matcuk et al. [5] did not describe MRI protocol nor statistical analysis with enough detail to be reproduced. Kitagawa et al. [16] did not report baseline characteristics for each treatment group separately. In addition, it was unclear if data was analysed in accordance with a pre-specified analysis plan.

The remaining seven studies carried a low risk of bias. Overall, given five studies exhibited some concerns or high risk of bias, and the presence of eight non-full text studies preventing assessment, the quality of evidence of studies included in this review was deemed as low.

### Hoffa’s fat pad composition

Zhong et al. [22] explored the relationship between fat content and composition in Hoffa’s fat pad and the severity of Hoffa synovitis in 64 knees with osteoarthritis. There was a good negative correlation (*r* = − 0.758) between fat fraction and severity of Hoffa-synovitis. Fat fraction was calculated as the ratio of fat within the pad to total amount of fat and water. The fat fraction decreased with worsening grade of Hoffa synovitis. There was no association between unsaturation index (ratio of unsaturated lipid to all lipids) and grade of Hoffa synovitis (*r* = − 0.152, *p* > 0.05).

Mikkilineni et al. [23] performed ultrasound imaging of 11 knees with Hoffa’s fat pad impingement and 10 asymptomatic controls before and after exercise. Following exercise, change in diameter of the largest vessel supplying the pad was greater, and trended toward dilation in asymptomatic knees compared to those with Hoffa’s fat pad impingement (*p* < 0.001). Compressibility of the fat pad before exercise was significantly lower in symptomatic knees compared to asymptomatic controls. There were no differences between groups in terms of subjective assessment of vascularity (*p* = 0.131), fat pad motion (*p* = 0.115), or percentage change of the largest fat lobule (*p* = 0.241), with respect to before and after exercise.

### Sports and Hoffa’s fat pad oedema

Yu [21] matched 31 knees of 29 professional football players who sustained football-related injuries to 31 outpatients with an acute knee injury. Characteristics of Hoffa’s fat pad under MRI were compared between the two groups. There was no statistically significant difference in the incidence of Hoffa’s fat pad oedema between both groups. However, incidence in both groups was not reported.

### Hoffa’s fat pad dimensions

von Engelhardt et al. [24] found patients with Hoffa’s fat pad impingement had a greater vertical and horizontal extent of Hoffa’s fat pad than those without. Kim et al. [25] compared radiographic parameters in 44 patients with Hoffa’s fat pad syndrome and 78 controls without knee pathology. They found no difference in Hoffa’s fat pad cross sectional area between patients with (695.23 mm^2^) and without (626.42 mm^2^) Hoffa’s fat pad syndrome (*p* > 0.05) [25].

### Bursae and fat pads related to Hoffa’s fat pad

von Engelhardt et al. [24] noted patients with Hoffa’s fat pad syndrome had a higher occurrence of a fluid-filled infrapatellar bursa (60% vs 43%). There was a significant association between the occurrence of deep infrapatellar bursitis and fat pad impingement (*p* = 0.016). There was a significant relation between Hoffa’s fat pad impingement and infra-hoffatic oedema within the superior aspect and apex of the fat pad (*p* < 0.001). These occurred more frequently in patients with Hoffa’s fat pad impingement than in those without (31% vs 11%). Bursae calcification and fibrosis were present more frequently in patients with Hoffa’s fat pad syndrome (7% vs 0.4%, and 18% vs 6%, respectively). von Engelhardt et al. [24] also noted that patients without Hoffa’s fat pad syndrome had a higher occurrence of joint effusion (71% vs 40%) and a higher prevalence of vertical (65% vs 44%) and horizontal (68% vs 48%) clefts. Lack of these clefts was associated with occurrence of Hoffa’s fat pad syndrome (*p* = 0.004).

### Patella-patellar tendon angle (PPTA)

Kim et al. [20] compared PPTA differences among patients with medial patellar plica (MPP) syndrome (*n* = 86), patellar chondromalacia (*n* = 44) and Hoffa’s fat pad syndrome (*n* = 26). The PPTA is the angle between a line connecting the upper and lower poles of the patella, and a line from the inferior aspect of the patella to the tibial tuberosity [20]. There was no significant difference in PPTA between patients. A direct comparison between patients with Hoffa’s fat pad syndrome and healthy controls was not performed [20]. Kim et al. [25] found PPTA was significantly lower in patients with Hoffa’s fat pad syndrome than in controls (137.3° vs 141.4°, respectively, *p* < 0.001).

### Patella alta

The Insall-Salvati ratio (ISR) is the ratio between the length of the patellar tendon and length of the patella [26]. A high ISR denotes a high-riding patella (patella alta). Seven studies found a high ISR was correlated with oedema in the superolateral portion of Hoffa’s fat pad [5, 18, 26–30].

There was no difference in ISR between patients with Hoffa’s fat pad syndrome (0.98) and healthy controls (1.00) in one study (*p* > 0.05) [25], van Middelkoop et al. [31] evaluated 133 patients with patellofemoral pain. Hoffa synovitis was present in 81. A large Insall-Salvati ratio was associated with the presence of Hoffa synovitis (odds ratio: 60.37).

### Trochlear angle

Trochlear angle is the angle between a line along the most anterior points of the medial and lateral trochlear facets, and the posterior cruciate ligament posterior condylar line [26]. Xiaolong et al. [30] evaluated MRIs of 60 patients with superolateral Hoffa’s fat pad oedema. Trochlear angle was positively correlated with oedema of the superolateral portion of Hoffa’s fat pad (*p* < 0.001). This was corroborated by Gürsoy et al. [18]. Widjajahakim et al. [26] evaluated MRI images of 1134 patients, of which 152 had superolateral Hoffa’s fat pad oedema. All patients were separated in quartiles according to degree of patellofemoral joint alignment and trochlear morphologic variables, with the lowest quartile approximating normal morphology more than the highest quartile. Superolateral Hoffa’s fat pad oedema was dichotomized as present or absent. Patients in the highest trochlear angle quartile (i.e. trochlear facet most anterior) had 1.6 times the odds of having superolateral Hoffa’s fat pad oedema than those in the lowest quartile.

### Sulcus angle

Sulcus angle is the angle between the medial and lateral trochlear facets [26]. Kim et al. [25] and van Middelkoop et al. [31] found no differences in sulcus angle between patients with and without Hoffa’s fat pad syndrome. Three studies found no association between sulcus angle and superolateral Hoffa’s fat pad oedema [5, 6, 26]. Kim et al. [29] found patients with superolateral Hoffa’s fat pad oedema had a wider sulcus angle than those without.

### Trochlear inclination

Kim et al. [25] found no difference in lateral trochlear inclination between patients with (23.7°) and without (24.3°) Hoffa’s fat pad syndrome (*p* > 0.05) (defined as the angle between the line connecting the most anterior points of the femoral condyle, and the line connecting the highest point of the lateral femoral trochlea and the deepest point of the trochlear groove). Widjajahakim et al. [26] and Mehta et al. [6] found no association between lateral trochlear inclination and superolateral Hoffa’s fat pad oedema. Matcuk et al. [5] found patients with superolateral Hoffa’s fat pad oedema had a lower lateral trochlear inclination of 22.1° than those without (24.4°).

### Epidemiological characteristics

Kim et al. [20] found no differences among patients with MPP syndrome, patellar chondromalacia and Hoffa’s fat pad syndrome in terms of patient age, sex, body mass index (BMI), and affected side of the knee. Campagna et al. [27] found younger patients were more likely to have superolateral Hoffa’s fat pad oedema, with age ranges not reported. Widjajahakim et al. [26] found no differences in prevalence of Hoffa’s fat pad oedema in different age and BMI groups. No studies evaluating relationship between ethnicity, employment, and Hoffa’s fat pad syndrome were identified.

### Tibial tubercle-tibial groove (TT-TG) distance

Six studies found a high TT-TG distance was associated with superolateral Hoffa’s fat pad oedema [5, 6, 25, 26, 28, 29].

### Patellar alignment and Hoffa’s fat pad syndrome:

Cilengir et al. [32] compared MRIs of 406 cases with a lateral patellar tilt angle (LPT) > 5° (group 1), and a control group of 40 cases with a LPT < 5° (group 2). Prevalence of Hoffa fat pad oedema was higher in group 1. A LPT of less than 10° was found to be a cut-off value to cause superolateral Hoffa fat pad oedema. van Middelkoop et al. [31] found Hoffa synovitis was not associated with patellar tilt, translation, patellar cartilage overlap, Wiberg classification (relating to degree of confluence between medial and lateral patellar facets), and bisect offset (percentage of the patella lateral to the line through the center of the trochlea). Widjajahakim et al. [26] found that knees with the highest bisect offset had 2.3 times the odds of having superolateral Hoffa’s fat pad oedema than those with the lowest. A strong direct relationship between increasing prevalence of superolateral Hoffa’s fat pad oedema and increasing quartile of bisect offset was observed. There was no association between patellar tilt angle and superolateral Hoffa fat pad oedema.

Kim et al. [25] compared MRI radiographic parameters in 44 patients with Hoffa’s fat pad syndrome and 78 controls without knee pathology. There were no differences between groups in axial patellar alignment and patellar tilt. Kim et al. [29] found patellofemoral maltracking was associated with superolateral Hoffa’s fat pad oedema. Gürsoy et al. [18] found patellofemoral angle was lower in patients with superolateral Hoffa’s fat pad syndrome. There were no differences between groups in terms of degree of patellar translation (defined as the distance between the most medial point of the patella to a line drawn at the most medial point of the femoral trochlea).

Campagna et al. [27] found short distance between patellar ligament and lateral trochlear facet were associated with superolateral Hoffa’s fat pad oedema. This could lead to impingement between the lateral femoral condyle and the posterior aspect of the patellar ligament. Mehta et al. [6] found patellar translation and prevalence of lateral patellar displacement were significantly higher in patients with superolateral Hoffa’s fat pad oedema. Patellofemoral angle was significantly lower in the superolateral Hoffa’s fat pad oedema group. There were no differences between the groups in terms of prevalence of lateral patellar tilt, mean number of abnormalities, and trochlear depth [6]. The latter is the mean of the maximum anteroposterior (AP) distance of the medial and lateral femoral condyles minus the AP distance between the deepest point of the trochlear groove and the line parallel to the posterior outline of the femoral condyles [28].

Matcuk et al. [5] found the following parameters were lower in patients with superolateral Hoffa’s fat pad oedema: length of medial patellar facet, patellar angle (angle between the medial and lateral facets of the patella), patellofemoral angle, patellar length, trochlear cartilage overlap (length of trochlear cartilage overlapping patellar cartilage) and trochlear cartilage index (ratio of trochlear cartilage overlap to patellar cartilage length). The following parameters were higher in patients with superolateral Hoffa’s fat pad oedema: length of medial and lateral trochlea, trochlear depth, lateral patellar displacement and tilt, and ventral trochlear prominence (greatest perpendicular distance between the antero-superior aspects of the femoral condyles). There was no difference between groups in terms of length of lateral facet of the patella, largest medial and lateral AP diameters of the femur, sulcus height (line perpendicular to posterior condylar axis, extending from midtrochlear groove) and patellar cartilage length [5]

Jibri et al. [28] evaluated anatomical parameters under MRI in 100 knees with superolateral Hoffa’s fat pad oedema (study group), and 100 knees with a normal Hoffa’s fat pad (control group). There was no statistically significant difference in trochlear depth between the groups. Patellar translation was significantly higher in patients with superolateral Hoffa’s fat pad oedema. Patellofemoral angle was significantly higher in the control group. In addition, there was a higher prevalence of lateral patellar displacement (> 2 mm patellar translation) and lateral patellar tilt in patients with superolateral Hoffa’s fat pad oedema. Overall, 60 patients in the study group had at least one abnormal patellar maltracking parameter in comparison to 16 in the control group.

Delorme and Jibri [33] matched 94 patients with patellar tendinosis (65 proximal and 29 distal tendinosis) with 94 controls. More patients had superolateral Hoffa’s fat pad impingement in the proximal tendinosis group than in the control group. No difference was observed between the distal patellar tendinosis group and control group.

### Potential management strategies for Hoffa’s fat pad syndrome

No studies exploring outcomes of treatment for Hoffa’s fat pad syndrome were published in the previous 10 years. Four studies reported on the effect of different interventions on Hoffa’s fat pad morphology. These were included as they could be potential management strategies for Hoffa’s fat pad syndrome. Kitagawa et al. [16] performed an RCT evaluating the effect of manual therapy or hot pack treatment on the flexibility of Hoffa’s fat pad in healthy subjects. Manual therapy consisted of pressing on the fat pad from its lateral aspect with a steady rhythm, for three minutes. The hot pack group received the intervention for 10 min. Results were compared to a control group who lied supine with their limbs relaxed for 10 min. The ratio of the thickness of the superficial part of the fat pad between 90° and 0° knee flexion was calculated to assess its flexibility. There was no difference between groups in terms of their effect on Hoffa’s fat pad flexibility.

Pogacnik Murillo et al. [19] randomised 454 overweight and obese adults with osteoarthritis into exercise only (E), diet-induced weight loss (D), and diet-induced weight loss + exercise (D + E). A subsample of 106 patients underwent MRI at baseline and 18-months follow-up, to analyse Hoffa’s fat pad volume, surface area, and thickness as part of a secondary analysis. There was, on average, 1.0% weight loss in E, 10.5% in D, and 13.0% in D + E. All groups experienced a reduction in fat pad volume (*p* < 0.01): E (− 2.1%), D (− 4.0%) and D + E (− 5.2%). All groups experienced a significant (*p* < 0.01) decrease in fat pad posterior surface area. Only the D + E group experienced a significant decrease in anterior surface area (*p* < 0.001). The D + E group demonstrated a greater reduction in fat pad volume and posterior surface area then the E group (*p* < 0.05). Other between-group differences in fat pad volume and surface area were not statistically significant. Across the three groups, there was a significant correlation between fat pad volume change and weight loss (*r* = 0.40, *p* < 0.01), total body fat mass change (*r* = 0.488), subcutaneous thigh fat change (*r* = 0.32), and inter-muscular thigh fat change (*r* = 0.29). On average, each percent of weight loss was related to a 0.27% reduction in fat pad volume.

Of 4796 patients in the osteoarthritis initiative, Steidle-Kloc et al. [34] evaluated 10 patients with > 20% weight gain, and nine patients with > 20% weight loss over 2 years. Fat pad volume was compared at baseline and at 2 years. Neither the patients with weight gain nor weight loss experienced statistically significant changes in fat pad volume.

Kalsi et al. [17] performed an RCT to evaluate the effectiveness of Tissue Gene-C (a TGF-Beta 1 expression vector) for the treatment of osteoarthritis. Its effects on Hoffa’s fat pad synovitis and effusion-synovitis at 12 months were amongst the secondary outcomes. When compared to placebo, Tissue Gene-C resulted in a lower progression rate (9.6% vs. 21.1%).

## Discussion

Current evidence suggests that high patellar height, TT-TG distance, and trochlear angle predispose the development of Hoffa’s fat pad syndrome. Eight studies found a high ISR (denoting a high-riding patella) was associated with Hoffa’s fat pad syndrome [5, 18, 26–31]. This was contradicted only by one study [25]. The concordance between multiple studies suggests patella alta is a risk factor for the development of Hoffa’s fat pad syndrome. Hoffa’s fat pad is an intra-capsular structure located inferior to the patella [1]. A high riding patella may apply traction on Hoffa’s fat pad, leading to oedema and fibrotic changes in the structure. Therefore, the evaluation of patella alta correction for treatment of Hoffa’s fat pad syndrome is warranted.

Six studies found a high TT-TG distance was associated with superolateral Hoffa’s fat pad oedema [5, 6, 25, 26, 28, 29]. A high TT-TG signifies a strong pull of the patellar tendon [35]. This could apply tension on Hoffa’s fat pad, resulting in its structural changes. A high trochlear angle was found to be associated with superolateral Hoffa’s fat pad oedema in three studies [18, 26, 30], suggesting this may be a risk factor for Hoffa’s fat pad syndrome. Similar to a high TT-TG distance leading to a strong pull of the patellar tendon, a high trochlear angle may be associated with altered patellar kinematics, leading to increased tension of Hoffa’s fat pad and resulting fibrosis. However, since this is hypothetical, further research to describe the causal relationship between high trochlear angle and Hoffa’s fat pad syndrome is required.

Three studies found trochlear inclination was not associated with Hoffa’s fat pad syndrome [6, 25, 26]. Five studies found sulcus angle was not associated with superolateral Hoffa’s fat pad oedema [5, 6, 25, 26, 31]. The concordance of multiple studies’ findings suggest these parameters are not risk factors for the development of Hoffa’s fat pad syndrome. This suggests surgical correction of trochlear inclination and sulcus angle for the treatment of Hoffa’s fat pad syndrome may not be warranted. Patient age and BMI were not associated with this condition either [20, 26]. Whether patient sex is a risk factor remains unclear due to conflicting evidence [20, 27]. No studies evaluating the relationship between Hoffa’s fat pad syndrome and ethnicity and employment were identified. Therefore, their impact on disease development cannot be established. Further research should stratify prevalence of Hoffa’s fat pad syndrome according to epidemiological characteristics to identify high risk groups and aid diagnosis.

There is a discrepancy in current evidence regarding the following potential risk factors for Hoffa’s fat pad syndrome, with differing findings in studies assessing them: Hoffa’s fat pad dimensions [24, 25], and measures of patellar alignment such as patellar tilt, inclination, translation, Wiberg classification and bisect offset [5, 6, 18, 25, 26, 28, 29, 31, 32]. Therefore, a link between these parameters and Hoffa’s fat pad syndrome cannot be reliably ascertained with current evidence.

This review identified high patellar height, TT-TG distance, and trochlear angle as predisposing factors for Hoffa’s fat pad syndrome. These constitute elements of altered patellar kinematics. Surgery aimed at correcting these may result in symptomatic relief. However, further research evaluating the effect of surgical intervention on Hoffa’s fat pad syndrome is required to corroborate this claim, since no studies reporting on this have been published in the last 10 years. Hoffa’s fat pad syndrome has a low incidence, and a low proportion of patients progress to surgery. Therefore, the performance of high-quality RCTs on the matter is unlikely. Improving our knowledge regarding surgical intervention may only be achieved by well-designed cohort studies [8]. Treating this condition remains challenging due to a lack of consensus regarding optimal management strategies. This is exacerbated by the lack of studies reporting on treatments for Hoffa’s fat pad syndrome. Four studies reported on the effect of different interventions on Hoffa’s fat pad morphology, but patients did not have Hoffa’s fat pad syndrome. Despite this, their findings may guide the development of management strategies for this condition. The application of hot packs or physiotherapy did not improve flexibility of Hoffa’s fat pad in healthy subjects [16]. However, further research evaluating manual therapies as a treatment for Hoffa’s fat pad syndrome should be conducted, since these could provide a non-invasive solution. Weight loss, achieved by either diet or exercise, resulted in a reduction of Hoffa’s fat pad volume and surface area [19]. Though this was contradicted by Steidle-Kloc et al. [34], their study had a lower sample size and level of evidence than the large RCT conducted by Pogacnik Murillo et al. [19]. This review identified high BMI was not a risk factor for developing Hoffa’s fat pad syndrome. However, weight loss may be beneficial once this has developed. Considering Hoffa’s fat pad syndrome is caused by fat pad impingement [2], a decrease in fat pad volume may result in symptomatic relief. Therefore, patients with Hoffa’s fat pad syndrome should be given advice on weight loss strategies. In addition to alleviating pain, patients are likely to benefit from overall improvements in health owing to weight loss [36]. Tissue Gene-C was found to lower progression rate of Hoffa’s fat pad synovitis in patients with osteoarthritis [17]. Since patients were not diagnosed with Hoffa’s fat pad syndrome, its ability to delay inflammation progression in this condition should be assessed. Studies reporting on stem cell therapies were excluded from data extraction in this review due to the pre-specified eligibility criteria of excluding animal studies. However, stem cell therapies have demonstrated the ability to reverse synovitis and fibrosis of Hoffa’s fat pad in animal models [10, 11]. Though the implementation of gene and stem cell therapies may be hindered by the high costs associated with these treatments, further study exploring their role in treating Hoffa’s fat pad syndrome is warranted. Continued research could result in cost-effective treatments for Hoffa’s fat pad syndrome.

Current evidence has limitations which must be improved upon to garner a better understanding of Hoffa’s fat pad syndrome. Firstly, multiple factors which could potentially predispose its development have been identified, but were reported by a single study. The lack of multiple studies exploring them hinders the validity of any conclusions drawn. These include low fat content in Hoffa’s fat pad [22], constriction of vessels supplying the fat pad, low fat pad compressibility [23], effusions and inflammation of the infrapatellar bursa [24], and PPTA [25]. Similarly, though the following factors were found not to predispose Hoffa’s fat pad syndrome, this cannot be corroborated due to being assessed in a single study: unsaturation index of Hoffa’s fat pad [22], vascularity, fat pad motion [23], playing sports [21], short distance between patellar ligament and lateral trochlear facet [25], and presence of patellar tendinosis [33]. Further research on parameters evaluated in a single study is required to corroborate whether they are risk factors for developing Hoffa’s fat pad syndrome. This is particularly relevant for sports, considering the high proportion of adolescents engaging in physical activity. Secondly, the link between Hoffa’s fat pad syndrome and other knee conditions should be established. Only one study evaluating this was identified [33]. Exploring the association between Hoffa’s fat pad syndrome and other common orthopaedic conditions such as osteoarthritis and patellar dislocation could help explain its pathophysiological processes. This could allow for the creation of tailored treatment regimens. Thirdly, no studies reporting on treatment for Hoffa’s fat pad syndrome were identified. Though results from studies reporting on Hoffa’s fat pad morphology following different interventions have been conducted, these do not provide a reliable appraisal of treatment for Hoffa’s fat pad syndrome. Further research should explore therapeutic strategies in patients with this condition. This review is limited by the inclusion of non-full text studies, in which risk of bias could not be assessed. The majority of studies included carried a low level of evidence and some concerns regarding risk of bias, which warrants caution when interpreting their findings. In addition, the heterogeneity of studies included prevented the performance of quantitative pooled analysis.

## Conclusion

Current evidence suggests that high patellar height, TT-TG distance, and trochlear angle predispose the development of Hoffa’s fat pad syndrome. In addition, trochlear inclination, sulcus angle, patient age and BMI do not seem to be associated with this condition. Further research should explore the link between Hoffa’s fat pad syndrome and sport as well as other conditions pertaining to the knee. In addition, further study evaluating treatment approaches for Hoffa’s fat pad syndrome is required.

## Data Availability

Not applicable

## Appendix 1: search strategy

Hoffa’s fat-pad OR Hoffa fat-pad OR infrapatellar fat pad OR IFP OR Hoffa’s fat pad syndrome OR Hoffa fat pad oedema

AND

Risk factors OR predisp* OR propens* OR risk OR prone OR patho* OR gender OR sex OR ethnicity OR race OR employment OR occupation OR job OR anatom* OR patell* OR radiograph* OR X-ray* OR MRI OR computed tomography OR CT OR ultrasound OR US OR treat* or management* or trial* or random* or RCT or randomized controlled trial OR interven* OR therapy OR medication* OR injection* OR arthroscopy OR surg OR platelet rich plasma OR steroids

Deduplicate

## Abbreviations

PRISMA: Preferred Reporting Items for Systematic Reviews and Meta-Analyses
TT-TG: Tibial Tubercle-Tibial Groove
BMI: Body Mass Index
EFORT: European federation of national associations of orthopaedics and traumatology
AQUA: Anatomical quality assessment
PPTA: Patella-patellar tendon angle
ISR: Insall-Salvati ratio
LPT: Lateral patellar tilt angle
AP: Anteroposterior
MRI: Magnetic Resonance Imaging
MR: Magnetic Resonance
NR: Not reported
NFT: Non-full text article

## References

[1] Gallagher J, Tierney P, Murray P, O’Brien M (2005). The infrapatellar fat pad: anatomy and clinical correlations. Knee Surg Sports Traumatol Arthrosc.

[2] Magi M, Branca A, Bucca C, Langerame V (1991). Hoffa disease. Ital J Orthop Traumatol.

[3] Larbi A, Cyteval C, Hamouri M, Dallaudiere B, Zarqane H, Viala P, Ruyer A (2014). Hoffa’s disease: a report on 5 cases. Diagn interv imaging.

[4] Dragoo JL, Johnson C, McConnell J (2012). Evaluation and treatment of disorders of the infrapatellar fat pad. Sports Med.

[5] Matcuk GR, Cen SY, Keyfes V, Patel DB, Gottsegen CJ, White EA (2014). Superolateral Hoffa fat-pad edema and patellofemoral maltracking: predictive modelling. Am J Roentgenol.

[6] Mehta K, Wissman R, England E, D’heurle A, Newton K, Kenter K (2015). Superolateral Hoffa’s fat pad edema in collegiate volleyball players. J Comput Assit Tomogr.

[7] Kim JH, Lee SK (2020). Superolateral Hoffa fat pad edema and patellofemoral maltracking: systematic review and meta-analysis. Am J Roentgenol.

[8] Rooney A, Wahba AJ, Smith TO, Donell ST (2015). The surgical treatment of anterior knee pain due to infrapatellar fat pad pathology: a systematic review. Orthop Traumatol Surg Res.

[9] Genin J, Faour M, Ramkumar PN, Yakubek G, Khlopas A, Chughtai M (2017). Infrapatellar fat pad impingement: a systematic review. J Knee Surg.

[10] Kouroupis D, Bowles AC, Best TM, Kaplan LD, Correa D (2020). CD10/neprilysin enrichment in infrapatellar fat pad-derived mesenchymal stem cells under regulatory-compliant conditions: implications for efficient synovitis and fat pad fibrosis reversal. Am J Sports Med.

[11] Kouroupis D, Willman MA, Best TM, Kaplan LD, Correa D (2021). Infrapatellar fat pad-derived mesenchymal stem cell-based spheroids enhance their therapeutic efficacy to reverse synovitis and fat pad fibrosis. Stem Cell Res Ther.

[12] Page MJ, McKenzie JE, Bossuyt PM, Boutron I, Hoffmann TC, Mulrow CD (2021). The PRISMA 2020 statement: an updated guideline for reporting systematic reviews. BMJ.

[13] Centre for Evidence-Based Medicine (2020) Oxford Centre for Evidence-Based Medicine: Levels of Evidence (March 2009). https://www.cebm.ox.ac.uk/resources/levels-of-evidence/oxford-centre-for-evidence-based-medicine-levels-of-evidence-march-2009. Accessed 17 Sep 2022.

[14] Henry BM, Tomaszewski KA, Ramakrishnan PK, Roy J, Vikse J, Loukas M (2016). Development of the Anatomical Quality Assessment (AQUA) tool for the quality assessment of anatomical studies included in meta-analyses and systematic reviews. Clin Anat.

[15] Sterne JAC, Savović J, Page MJ, Elbers RG, Blencowe NS, Boutron I (2019). RoB 2: a revised tool for assessing risk of bias in randomised trials. BMJ.

[16] Kitagawa T, Ozaki N, Aoki Y (2022). Effect of physical therapy on the flexibility of the infrapatellar fat pad: a single-blind randomised controlled trial. PLoS ONE.

[17] Kalsi G, Parvizi J, Bramlet D, Romness D, Guermazi A, Noh M (2018). The efficacy and safety of genetically engineered allogeneic human chondrocytes expressing TGF-beta1 in patients with grade 3 chronic degenerative joint disease of the knee.

[18] Gürsoy M, Mete BD, Oyar O, Erdoğan N, Uluç ME, Bulut T, Gürsoy S (2018). The association of patellar maltracking with infrapatellar fat pad edema and chondromalacia patella: a quantitative morphological magnetic resonance imaging analysis. Turk J Phys Med Rehabil.

[19] Pogacnik Murillo AL, Eckstein F, Wirth W, Beavers D, Loeser RF, Nicklas BJ (2017). Impact of diet and/or exercise intervention on infrapatellar fat pad morphology: secondary analysis from the intensive diet and exercise for arthritis (IDEA) trial. Cells Tissues Organs.

[20] Kim T, Kim JK, Lee HS, Kim DK (2022). Patella-patellar tendon angle in relation to the medial patellar plica syndrome, chondromalacia patella, and infrapatellar fat pad syndrome. PLoS ONE.

[21] Yu W (2022). Observation on the effect of MRI image scanning on knee pain in football injury. Scanning.

[22] Zhong L, Li M, Du X, Ding Y, Zhang X, Mei Y, Yi P, Feng Y, Chen Y, Zhang X (2022). Quantitative evaluation of the characteristic of infrapatellar fat pad fat content and unsaturation index by using hydrogen proton MR spectroscopy. Magn Reson Imaging.

[23] Mikkilineni H, Delzell PB, Andrish J, Bullen J, Obuchowski NA, Subhas N, Polster JM, Schils JP (2018). Ultrasound evaluation of infrapatellar fat pad impingement: an exploratory prospective study. Knee.

[24] von Engelhardt LV, Tokmakidis E, Lahner M, Dàvid A, Haage P, Bouillon B, Lichtinger TK (2020). Hoffa’s fat pad impingement treated arthroscopically: related findings on preoperative MRI in a case series of 62 patients. Arch Orthop Trauma Surg.

[25] Kim YM, Joo YB, Lee WY, Park IY, Park YC (2020). Patella-patellar tendon angle decreases in patients with infrapatellar fat pad syndrome and medial patellar plica syndrome. Knee Surg Sports Traumatol Arthrosc.

[26] Widjajahakim R, Roux M, Jarraya M, Roemer FW, Neogi T, Lynch JA (2017). Relationship of trochlear morphology and patellofemoral joint alignment to superolateral hoffa fat pad edema on MR images in individuals with or at risk for osteoarthritis of the knee: the MOST study. Radiology.

[27] Campagna R, Pessis E, Biau DJ, Guerini H, Feydy A, Thevenin FS, Pluot E, Rousseau J, Drapé JL (2012). Is superolateral Hoffa fat pad edema a consequence of impingement between lateral femoral condyle and patellar ligament?. Radiol.

[28] Jibri Z, Martin D, Mansour R, Kamath S (2012). The association of infrapatellar fat pad oedema with patellar maltracking: a case-control study. Skeletal Radiol.

[29] Kim JH, Lee SK, Jung JY (2019). Superolateral Hoffa’s fat pad oedema: relationship with cartilage T2* value and patellofemoral maltracking. Eur J Radiol.

[30] Xiaolong C, Heng Z, Rong H, Guanghua L, Jincai L (2021). Correlation of infrapatellar fat pad edema with trochlear and patellofemoral joint morphology: MRI evaluation. Chin J Tissue Eng Res.

[31] van Middelkoop M, Macri EM, Eijkenboom JF, van der Heijden RA, Crossley KM, Bierma-Zeinstra SMA (2018). Are patellofemoral joint alignment and shape associated with structural magnetic resonance imaging abnormalities and symptoms among people with patellofemoral pain?. Am J Sports Med.

[32] Cilengir AH, Cetinoglu YK, Kazimoglu C, Gelal MF, Mete BD, Elmali F, Tosun O (2021). The relationship between patellar tilt and quadriceps patellar tendon angle with anatomical variations and pathologies of the knee joint. Eur J Radiol.

[33] Delorme JP, Jibri Z (2021). The association of patellar tendinosis with patellar maltracking and Hoffa’s fat pad impingement: a case-control MRI study. Clin Imaging.

[34] Steidle-Kloc E, Dannhauer T, Wirth W, Echkstein F (2015). Responsiveness of infra-patellar fat pad (IPFP) volume change with severe body weight gain and loss—data from the OAI. Osteoarthr Cartil.

[35] Carlson VR, Boden BP, Shen A, Jackson JN, Yao L, Sheehan FT (2017). The tibial tubercle-trochlear groove distance is greater in patients with patellofemoral pain: implications for the origin of pain and clinical interventions. Am J Sports Med.

[36] Blackburn G (1995). Effect of degree of weight loss on health benefits. Obes Res.

